# Digital Interventions to Promote Health and Well-Being of Older Adults

**DOI:** 10.1093/geroni/igaa057.2721

**Published:** 2020-12-16

**Authors:** Gloria Gutman, Marcia Shade

## Abstract

The population of older adults is on the rise and so is their adoption of technology. According to the Pew Research Center, growing numbers of older adults are using smartphones, tablets, and the internet. Technology can also be an innovative and accessible way to deliver interventions that promote positive health outcomes. But implementing and evaluating digital interventions, especially those targeted to cognitively frail or otherwise vulnerable older adults, is not easy. The first presentation in this international interdisciplinary symposium sets the stage for discussing some of the barriers encountered. Specifically, it addresses the issue of compliance of adults with Mild Cognitive Impairment (MCI) while using internet-based cognitive behavioral therapy for sleep disturbance. The second presentation described a digital tool to screen and interview older adults in a hospital emergency department who may be experiencing mistreatment. This is followed by a study of use of voice assistant reminders to help aging adults adhere to daily completion of a diary to self-monitor pain symptoms. The fourth presentation describes a feasibility study of a digital screen-based device to calm long-term care home residents exhibiting disruptive Behavioral and Psychological Symptoms of Dementia. Symposium speakers and participants will be encouraged to dialogue on how some of the implementation and evaluation barriers described might be overcome or avoided as well as to share success stories from their own research on digital interventions for older adults living at home, in congregate housing or in hospital settings.

